# Upadacitinib for refractory generalized lichen sclerosus: a case report and brief literature review

**DOI:** 10.3389/fimmu.2026.1873990

**Published:** 2026-07-01

**Authors:** Mengqiu Xiao, Jingyi Li, Zhendong Wei, Kun Qu, Rongxin Zhang, Aoxue Wang

**Affiliations:** Department of Dermatology, The Second Hospital of Dalian Medical University, Dalian, Liaoning, China

**Keywords:** generalized lichen sclerosus, upadacitinib, JAK1 inhibitor, review, case report

## Abstract

Lichen sclerosus (LS) is a chronic, lymphocyte-mediated inflammatory skin disease characterized by porcelain-white atrophic skin lesions. Localized LS is relatively common and predominantly occurs in the anogenital region. Generalized LS involving both genital and extragenital sites is clinically rare and frequently refractory to conventional therapies like topical corticosteroids. As an emerging targeted therapy, Janus kinase (JAK) inhibitors demonstrate broad therapeutic potential. The highly selective JAK1 inhibitor upadacitinib demonstrates robust efficacy across various inflammatory dermatoses; however, reports of its application in LS remain scarce. Herein, we report the case of a 62-year-old female patient with refractory generalized LS who presented with a 2-year course. The skin lesions involved the trunk, extremities and vulvar region, and failed to respond to multiple conventional treatments. Upadacitinib at a dose of 15 mg daily was administered to the patient. During the 10-month follow-up period, pruritus was significantly alleviated, and skin sclerosis as well as atrophic patches showed marked improvement. No new blisters or skin lesions emerged, and no severe adverse reactions were observed. This case supports the effectiveness of upadacitinib in refractory generalized LS and offers a brief review of relevant literature.

## 1 Introduction

Lichen sclerosus (LS) is a chronic, lymphocyte-mediated inflammatory skin disease clinically characterized by porcelain-white atrophic skin lesions. The disease predominantly affects the anogenital region, whereas generalized LS involves both genital and extragenital sites, with extragenital involvement appearing on the trunk and extremities. Patients typically present with extensive lesions and severe pruritus, profoundly impairing their quality of life. Generalized LS is relatively rare and more challenging to treat. Its typical histological alterations include epidermal atrophy, homogenization or hyalinization of dermal collagen, lymphocytic infiltration, and may be accompanied by liquefactive degeneration of the basement membrane zone and dermal edema (1–5).

Currently, first-line treatment regimens primarily consist of potent topical corticosteroids (TCS) and topical calcineurin inhibitors (TCI). However, for generalized LS, local treatment is often limited by the application area and patient compliance. Although systemic immunosuppressants may serve as second-line options, their efficacy varies, and they may carry significant toxic side effects (1, 2, 6). Consequently, exploring targeted therapeutic strategies for specific immune pathways holds significant clinical implications. Studies indicate that LS is predominantly induced by mechanisms such as autoimmune dysregulation, oxidative stress and genetic mutations, among which the T helper 1 (Th1)-type immune response and interferon-gamma (IFN-γ) signaling play pivotal roles. These signals are highly dependent on the Janus kinase-signal transducer and activator of transcription (JAK-STAT) pathway. Theoretically, JAK inhibitors can exert therapeutic effects by intervening at relevant stages (1, 4, 6). Given the established clinical application of JAK inhibitors in immune-mediated inflammatory dermatoses such as psoriatic arthritis and atopic dermatitis (7), their potential efficacy in LS has gradually attracted attention. In the current literature, evidence regarding JAK inhibitors for LS is primarily limited to single-arm studies and case reports. Clinical evidence concerning the use of upadacitinib for refractory generalized LS remains particularly scarce. This article reports a case of refractory generalized LS successfully treated with upadacitinib, a highly selective JAK1 inhibitor, and provides a brief review of the existing literature to offer clinical insights for such challenging cases.

## 2 Case presentation

**Figure 1:**
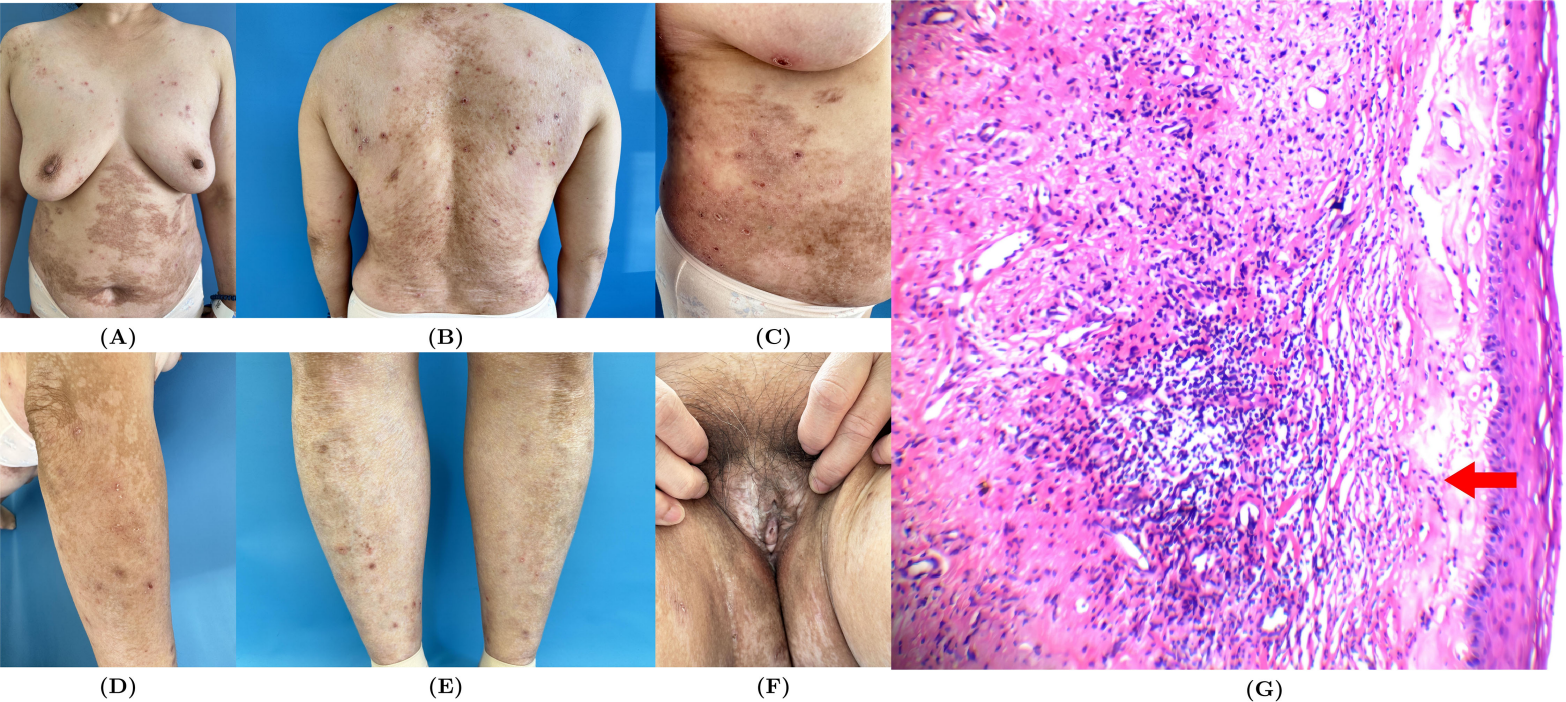
Generalized LS lesions on the chest and abdomen **(A)**, back **(B)**, right abdomen **(C)**, right upper limb **(D)**, anterior calves **(E)**, and perineum **(F)**. Histopathological features of the abdominal lesion are shown in **(G)** (H&E stain, ×50), revealing prominent epidermal hyperkeratosis and significant atrophy of the spinous layer, a broad zone of hyalinization and homogenization of the papillary dermis (red arrow), and a band-like mononuclear cell infiltrate in the superficial dermis.

A 62-year-old female patient presented with generalized atrophic patches accompanied by pruritus for 1.5 years, which worsened and was accompanied by pain for 2 months. Two years ago, prior to the generalized skin lesions, the patient initially presented with vulvar atrophy. One and a half years ago, the lesions spread to the trunk and extremities, manifesting as hyperpigmentation, skin sclerosis, atrophic patches and even blisters, accompanied by significant pruritus. A skin biopsy performed at another hospital confirmed the diagnosis of LS. She was refractory to multiple systemic and topical interventions, including antihistamines, thalidomide, traditional Chinese medicine, topical corticosteroids, and tacrolimus. She had no history of infectious diseases, immunological disorders, or tumors, but she was allergic to hydroxychloroquine. Physical examination revealed well-demarcated porcelain-white atrophic patches with a glossy surface scattered over the trunk, extremities and external genitalia. The trunk exhibited numerous yellowish-brown flat papules and plaques with some areas showing ulcerated and crusted surfaces. No involvement of the oral mucosa was observed (**Figures 1A–F**).

**Figure 2:**
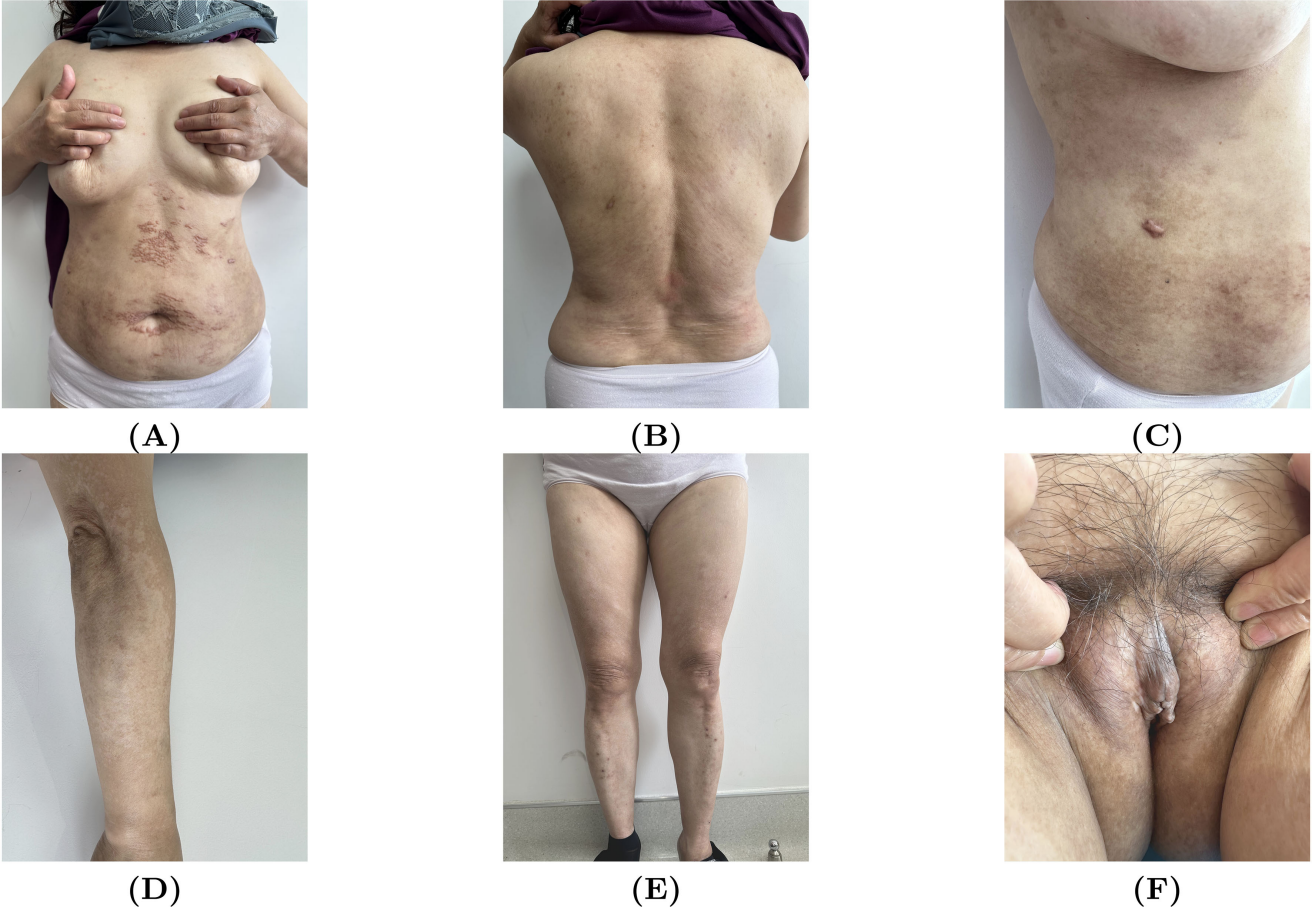
Post-treatment clinical photographs at the 10-month follow-up after upadacitinib 15 mg once daily, showing clinical improvement of generalized LS on the chest and abdomen **(A)**, back **(B)**, right abdomen **(C)**, right upper limb **(D)**, anterior calves **(E)**, and perineum **(F)**.

**Figure 3:**
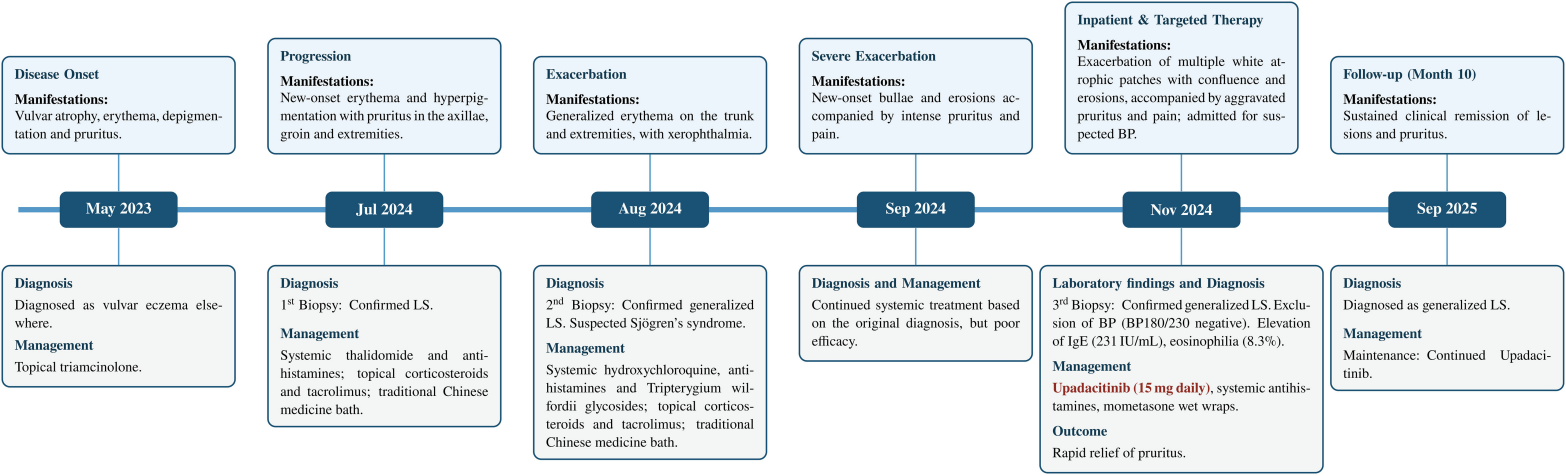
Clinical timeline illustrating disease progression and therapeutic response. Generalized lichen sclerosus was definitively diagnosed through three successive biopsies after the failure of conventional systemic therapies. Intervention with upadacitinib (15 mg daily) induced rapid and durable clinical remission. BP, bullous pemphigoid; IgE, immunoglobulin E; JAK1, Janus kinase 1; LS, lichen sclerosus.

Laboratory investigations revealed an elevated eosinophil percentage of 8.3% (reference range: 0.4%–8.0%). Inflammatory and immune-related parameters, including interleukin (IL)-6 (3.55 U/mL), IL-8 (188 U/mL), tumor necrosis factor-alpha (TNF-*α*) (11.6 pg/mL), and immunoglobulin E (IgE) (231 IU/mL), were all significantly elevated. The antibody levels for BP180 and BP230, C-reactive protein, erythrocyte sedimentation rate (ESR), hepatitis and tuberculosis testing, the autoimmune antibody profile and chest CT scan were all within normal ranges. Abdominal ultrasound revealed mild fatty liver and right renal cyst. Histopathological examination of the abdominal lesion exhibited hyperkeratosis, parakeratosis, thickening of the granular layer, irregular acanthosis, vacuolar degeneration of focal basal cells, marked homogenization of collagen fibers in the papillary dermis, and a perivascular lymphocytic infiltrate accompanied by abundant eosinophils in the superficial dermis (**Figure 1G**). The diagnosis was confirmed as generalized LS based on the patient's skin lesions of pruritic atrophic patches, plaques and sclerosis, with negative serum BP180 and BP230 antibodies and characteristic histopathological findings. Bullous pemphigoid, lichen planus and chronic eczema were excluded. She presented with a prolonged disease course, extensive distribution of skin lesions, severe pruritus, and poor response to multiple conventional treatment regimens, which aligns with the clinical characteristics of refractory LS. The patient was administered upadacitinib 15 mg once daily, together with ebastine 10 mg nightly and olopatadine 5 mg twice daily. For acute inflammation control, mometasone furoate cream was applied once daily to the abdominal lesions using wet-wrap therapy. The patient experienced rapid relief of pruritus, followed by gradual improvement of the skin lesions. After 4 weeks of treatment, the VAS score decreased from a baseline value of 9 to 1. At the 10 months follow-up, significant improvement was observed in the atrophic patches, papules, plaques, blisters, crusts and skin dryness, with VAS score decreased to 0 (**Figures 2A–F**). During follow-up, routine blood tests, liver and kidney function tests, and lipid profiles showed no significant abnormalities, with no recurrence of skin lesions and a marked improvement in quality of life. To illustrate the clinical trajectory, **Figure 3** provides a comprehensive timeline detailing the treatment sequence and disease progression.

## 3 Discussion

LS is a chronic inflammatory dermatosis that most commonly affects the anogenital region. It is classically characterized by porcelain-white atrophic patches with pruritus or pain. Prolonged disease course may lead to scarring, functional impairment, and increased risk of squamous cell carcinoma. Compared to the localized variant, generalized LS is significantly less common and frequently involves both genital and extragenital sites simultaneously. Lesion morphology can manifest as atrophic patches, papules, vesicles, and poikilodermatous changes. Its clinical resemblance to eczema, psoriasis, lichen planus, morphea, and autoimmune bullous dermatoses frequently delays accurate diagnosis and appropriate treatment. Consequently, for patients presenting with extensive lesions, atypical manifestations or suboptimal therapeutic responses, early multi-site biopsies remain crucial for improving diagnostic accuracy (1, 6).

The pathogenesis of generalized LS involves complex interactions of multiple factors including immune dysregulation, oxidative stress and genetic mutations (5). Conventionally, LS is considered to be primarily driven by a T helper 1 (Th1)-type immune response. IFN-γ upregulates the expression of chemokines such as CXCL9 and CXCL10, recruiting cytotoxic T cells to attack basal keratinocytes through the JAK-STAT signaling pathway, ultimately leading to basement membrane damage, abnormal collagen metabolism and persistent inflammation (5, 8–10).

The clinical features of the patient with refractory generalized LS in this case suggest a more complex immunological pathogenesis. The patient’s pre-treatment elevated serum IgE and peripheral eosinophilia may indicate a concurrent Th2-skewed inflammatory profile. LS is traditionally considered to be driven primarily by a Th1/IFN-γ-mediated immune response. However, given previous studies linking LS with atopic conditions, the present findings raise the possibility that the generalized refractory subtype reflects a mixed immune dysregulation model which remains Th1-driven but features concurrent Th2 involvement. This mixed phenotype may partially explain the protracted disease course, prominent pruritus, and inadequate response to conventional therapies. However, this hypothesis still requires further validation through higher-level studies (11).

The JAK-STAT pathway is a central cascade in intracellular signal transduction that mediates the inflammatory signaling of multiple cytokines (12), and is implicated in various immune-mediated dermatoses, such as psoriasis and atopic dermatitis (7). By intervening in the JAK-STAT signaling pathway, JAK inhibitors can block multiple inflammatory cascades upstream, thereby potentially alleviating pruritus, inflammatory infiltration and the subsequent sclerotic process. This underscores the potential therapeutic efficacy of JAK inhibitors in LS. The unique mixed immune phenotype observed in this patient provides a plausible mechanistic explanation for the remarkable efficacy of the selective JAK1 inhibitor, upadacitinib. Unlike conventional agents, upadacitinib achieves a “dual coverage” of key pathogenic pathways through JAK1 inhibition: 1) Blocking the Th1 axis: It suppresses IFN-*γ*, which relies on JAK1/JAK2 signaling, thereby preventing cytotoxic damage and attenuating classic lichenoid histological alterations and the sclerotic process (13); 2) Blocking the Th2 axis: It effectively inhibits JAK1-dependent Th2-type cytokines (such as IL-4 and IL-13), as well as IL-31 and its receptor signaling, which are closely associated with severe pruritus (14–16). Following upadacitinib administration, the patient experienced rapid alleviation of pruritus and significant improvement in skin lesions. This suggests that for patients with LS accompanied by atopic features or extensive skin lesions, targeting JAK1 may be more valuable for the treatment of LS compared to conventional immunosuppressants.

To date, there have been 12 publications on the role of JAK inhibitors in the treatment of LS, primarily consisting of case reports, small-sample studies and limited prospective studies, covering various targets and administration methods, as shown in **Table 1**. Current studies include both oral and topical formulations, focusing primarily on baricitinib (a JAK1/2 inhibitor), tofacitinib (which primarily inhibits JAK1/3), and abrocitinib (a selective JAK1 inhibitor). Baricitinib has demonstrated favorable clinical feasibility and tolerability in improving adult genital LS (GLS), generalized extragenital involvement, and pediatric extragenital cases (17–20). Abrocitinib has exhibited distinct advantages in achieving rapid pruritus relief and short-term remission in refractory GLS (21, 22). Tofacitinib can significantly ameliorate severe LS complicated by bullous lesions and generalized morphea overlap syndrome (23). Furthermore, in a retrospective case series study, tofacitinib showed remarkable efficacy in adult GLS, and has also been successfully used in pediatric cases of generalized LS (8). Although topical ruxolitinib cream provides an alternative for localized skin lesions and sensitive areas (e.g., the vulva and in pediatric patients) (24, 25), systemic intervention remains the preferred option for extensive LS with widespread involvement of multiple sites and resistance to conventional therapies.

**Table 1:** Overview of reported evidence for JAK inhibitors in the treatment of lichen sclerosus (LS).

Author	Agent	Dosage and duration	LS Type/location	Main outcomes	Safety/adverse events	Evidence type
Li et al., 2021 (18)	Baricitinib	2 mg daily + PUVA (twice weekly) for 3 months	EGLS (trunk, lower extremities)	Significant repigmentation, restored skin elasticity, and softened sclerotic plaques at 3 months; improved quality of life.	None reported	Case report (n=1)
Su et al., 2022 (20)	Baricitinib	2 mg daily for 6 months	Pediatric EGLS (right neck)	Marginal repigmentation and reduced induration at 2 months; 50% lesion repigmentation at 6 months	None reported	Case report (n=1)
Wang et al., 2023 (19)	Baricitinib	2 mg daily for 3 months	Vaccine-induced EGLS (trunk, extremities)	Skin induration was significantly alleviated, and inflammatory erythema resolved at 3 months; no recurrence	None reported	Case report (n=1)
Bao et al., 2023 (17)	Baricitinib	4 mg daily for 6 months	GLS	100% achieved disease control (IGA ≤ 1 with ≥ 2-grade improvement) at 20 weeks; improved pruritus, pain, and quality of life	Well-tolerated; mild infections and transient lab abnormalities	Single-arm prospective study (n=26)
Bao et al., 2023 (21)	Abrocitinib	100 mg daily for 4 months	GLS	Rapid pruritus relief; IGA control at 12 weeks	Mild, transient elevations in platelets/cholesterol (n=1)	Single-arm study (n=10)
Xiong et al., 2024 (22)	Abrocitinib	100 mg daily for 6 months	MGLS with plasma cell balanitis	Erythema/erosions improved by day 3; complete remission at 1 month; no recurrence at 6 months.	None reported	Case report (n=1)
Jiang et al., 2023 (24)	Ruxolitinib	1.5% cream BID for 8 months	EGLS (Fitzpatrick IV–V)	Decreased plaque firmness and markedly reduced pruritus and tenderness at 2 months	None reported (continuous skin cancer screening advised)	Case series (n=3; 1 treated with topical JAK inhibitor)
Zundell et al., 2024 (25)	Ruxolitinib	1.5% cream BID for 6 weeks	Pediatric GLS (vulvar/perianal)	Significant erythema reduction; resolved pruritus and dysuria at 6 weeks	None reported	Case series (n=4; 1 with LS)
Liu et al., 2022 (23)	Tofacitinib	5 mg BID for 4 months (discontinued due to concurrent diffuse large B-cell lymphoma)	Bullous LS-generalized morphea overlap	Ulcer healing at 4 weeks; reduced induration and improved knee contractures at 4 months. Relapse upon discontinuation	Developed diffuse large B-cell lymphoma (causal relationship “undetermined”)	Case report (n=1)
Sharma et al., 2025 (8)	Tofacitinib	5 mg daily for 6 months	Pediatric generalized LS (anogenital, chest, lower extremities)	Resolved pruritus and dyschezia; partial repigmentation (from depigmentation to hypopigmentation); dermoscopic resolution of follicular plugging	None reported	Case report (n=1)
Shao et al., 2026 (27)	Tofacitinib	11 mg daily for 12 weeks	GLS	Week 4: IGA ≤1 (38.5%), NRS clearance (53.8%), Dysuria resolved in 85.7% of affected patients. Week 12: IGA ≤1 & NRS clearance (92.3%), DLQI clearance (69.2%), Baseline dyspareunia/tenderness resolved (61.5%). Follow-up: No recurrence during 1–13 months of post-treatment follow-up	Back folliculitis (n=1)	Retrospective case series (n=13)
Cao et al., 2025 (26)	Upadacitinib	15 mg daily for 6 months	LS-morphea overlap (hands)	Softened lesions and reduced pain at 3 weeks; almost complete resolution at 6 months	None reported	Case report (n=1)
Present case	Upadacitinib	15 mg daily for 10 months	Refractory generalized LS	Rapid pruritus resolution; sustained improvement in atrophic plaques and induration	Mild, asymptomatic lipid elevation	Case report (n=1)

LS, lichen sclerosus; PUVA, psoralen and ultraviolet A; GLS, genital lichen sclerosus; IGA, Investigator’s Global Assessment; MGLS, male genital lichen sclerosus; BID, twice daily; EGLS, extragenital lichen sclerosus; NRS, Numeric Rating Scale; DLQI, Dermatology Life Quality Index.

Although the pivotal role of the JAK pathway in the pathogenesis of LS has been widely recognized, studies on the treatment of LS with the highly selective JAK1 inhibitor upadacitinib remain limited to case reports. In 2025, Cao et al. reported a 53-year-old female patient with an LS-morphea overlap syndrome, following 6 months of treatment with upadacitinib (15 mg daily), the skin lesions on her hands almost completely resolved and the regimen was well tolerated (26). However, the lesions in that patient were localized. To date, there have been no documented case reports regarding the use of upadacitinib extended-release tablets for the treatment of generalized LS.

The skin lesions in this patient manifested as atrophic patches, papules, blisters, and poikilodermatous changes, indicative of refractory generalized LS in adults. The prolonged disease course and severe pruritus are prone to misdiagnosis, leading to delayed standardized treatment. Following upadacitinib therapy, the patient exhibited sustained improvement in both genital and extragenital lesions, with significant relief of pruritus and no recurrence. The patient exhibited no significant adverse reactions during treatment demonstrating good safety profile. This highlights the therapeutic potential of upadacitinib in generalized LS and provides a novel treatment direction for this refractory case. However, studies of larger sample sizes and controlled trials are needed to further confirm the efficacy of upadacitinib.

## 4 Conclusion

LS is an immune-driven, chronic inflammatory dermatosis associated with oxidative stress and genetic susceptibility. The condition typically presents with porcelain-white atrophic plaques and intense pruritus. Key histological features include epidermal atrophy, homogenized dermal collagen and perivascular lymphocytic infiltrates. While localized anogenital LS is common, generalized LS involving both genital and extragenital sites is rare and often refractory to topical corticosteroids. Conventional topical therapies also face limitations, such as poor patient adherence, persistent skin induration and the risk of long-term steroid-induced atrophy. Meanwhile, systemic immunosuppressants are often limited by potential toxicity. Recently, JAK inhibitors have emerged as promising targeted therapies due to their convenient dosing and favorable safety profiles. In particular, the selective JAK1 inhibitor upadacitinib has shown robust efficacy in various immune-mediated diseases. However, its application in LS remains rarely reported.

We report the case of a 62-year-old female with refractory generalized LS, highlighting upadacitinib as a highly effective and well-tolerated systemic alternative. By modulating key pathogenic immune pathways, upadacitinib offers a promising therapeutic strategy for complex cases resistant to conventional therapies. Nevertheless, large-scale, controlled clinical trials are warranted to establish optimal dosing, long-term safety and definitive efficacy across diverse patient populations.

## Data Availability

The original contributions presented in the study are included in the article/supplementary material. Further inquiries can be directed to the corresponding author.
